# Atypical role of sprouty in colorectal cancer: sprouty repression inhibits epithelial–mesenchymal transition

**DOI:** 10.1038/onc.2015.365

**Published:** 2015-10-05

**Authors:** Q Zhang, T Wei, K Shim, K Wright, K Xu, H L Palka-Hamblin, A Jurkevich, S Khare

**Affiliations:** 1 Department of Internal Medicine, Section of Gastroenterology and Hepatology, University of Missouri, Columbia, MO, USA; 2 Department of Pediatrics, Medical College of Wisconsin, Milwaukee, WI, USA; 3 Department of Biochemistry and Molecular Genetics, University of Illinois at Chicago, Chicago, IL, USA; 4 Molecular Cytology Core, University of Missouri, Columbia, MO, USA; 5 Harry S. Truman Memorial Veterans' Hospital, Columbia, MO, USA

## Abstract

Sprouty (SPRY) appears to act as a tumor suppressor in cancer, whereas we demonstrated that SPRY2 functions as a putative oncogene in colorectal cancer (CRC) (Oncogene, 2010, 29: 5241–5253). We investigated the mechanisms by which SPRY regulates epithelial–mesenchymal transition (EMT) in CRC. SPRY1 and SPRY2 mRNA transcripts were significantly upregulated in human CRC. Suppression of SPRY2 repressed AKT2 and EMT-inducing transcription factors and significantly increased E-cadherin expression. Concurrent downregulation of SPRY1 and SPRY2 also increased E-cadherin and suppressed mesenchymal markers in colon cancer cells. An inverse expression pattern between AKT2 and E-cadherin was established in a human CRC tissue microarray. SPRY2 negatively regulated miR-194-5p that interacts with AKT2 3′ untranslated region. Mir-194 mimics increased E-cadherin expression and suppressed cancer cell migration and invasion. By confocal microscopy, we demonstrated redistribution of E-cadherin to plasma membrane in colon cancer cells transfected with miR-194. *Spry1*^*−/−*^ and *Spry2*^*−/−*^ double mutant mouse embryonic fibroblasts exhibited decreased cell migration while acquiring several epithelial markers. In CRC, SPRY drive EMT and may serve as a biomarker of poor prognosis.

## Introduction

Sprouty (SPRY) is an intracellular regulator of receptor tyrosine kinase signaling involved in growth, differentiation and tumorigenesis. Four family members of SPRY (SPRY1–4) have been identified.^1, 2^ SPRY2 appears to be ubiquitously expressed, whereas other family members show organ and tissue specificity.^3^ Experimental evidence demonstrate that SPRY specifically inhibits activation of extracellular-regulated kinase in response to nerve growth factor, platelet-derived growth factor (PDGF), vascular endothelial growth factor, brain derived neutrotrophic factor, glial cell line-derived neutrotrophic factor and fibroblast growth factor.^4^ On the contrary, mitogen-activated protein kinase activation is not always inhibited by SPRY. Surprisingly, in some instances, SPRY1 and SPRY2 not only failed to suppress epidermal growth factor-induced mitogen-activated protein kinase activation but also enhanced activation of this pathway.^4^

Expression of SPRY1 and SPRY2 is reduced in breast, prostate, lung and liver carcinoma, suggesting a tumor-suppressor role of SPRY in cancer.^4^ Role of SPRY in colorectal cancer (CRC) is still evolving. We demonstrated increased SPRY2 protein expression in human CRC.^5^ Likewise, high expression of SPRY2 in human CRC and its association with poor patient survival has been recently reported.^6^ On the contrary, other investigators noted reduced SPRY2 transcripts in intestinal tumors.^7^ Interestingly, a meta-analysis at the oncomine database concluded that SPRY2 expression is higher in CRC tumors than in other cancers.^8^ An inverse correlation of SPRY2 with E-cadherin and increased immunoreactive SPRY2 in undifferentiated high-grade tumors and at the invasive front of low-grade carcinomas implicated SPRY2 in tumor metastasis.^9^ The study of gene expression signatures in CRC with or without *KRAS* mutations also revealed upregulation of SPRY2 in *KRAS* mutant tumors.^10^ Mechanistically, we established that TAT-SPRY2 protein transduction or SPRY2 cDNA stable transfection significantly increased tumorigenicity and metastatic potential of colon cancer cells via elevated c-Met expression.^5^

Despite these observations, molecular regulation of epithelial-to-mesenchymal transition (EMT) by SPRY2 is never investigated in CRC. In this study, we provided the experimental evidence that SPRY2 regulates miR-194. SPRY2 and miR-194-dependent suppression of AKT2 and other repressors of E-cadherin may account for upregulation of E-cadherin and inhibition of cancer cell migration and invasion. Underscoring the biological relevance of these observations, recombination of floxed SPRY1 and SPRY2 alleles in mouse embryonic fibroblasts (MEFs) resulted in increased expression of epithelial markers and decreased cell migration. Further, expression profile analysis in human CRC revealed increased SPRY1 and SPRY2 transcripts and an inverse expression pattern between AKT2 and E-cadherin. Together, this study indicates that SPRY is a target of therapeutic intervention in CRC metastasis.

## Results

### SPRY is upregulated in human CRC and positively regulates metastatic potential of colon cancer cells

**Figure 1:**
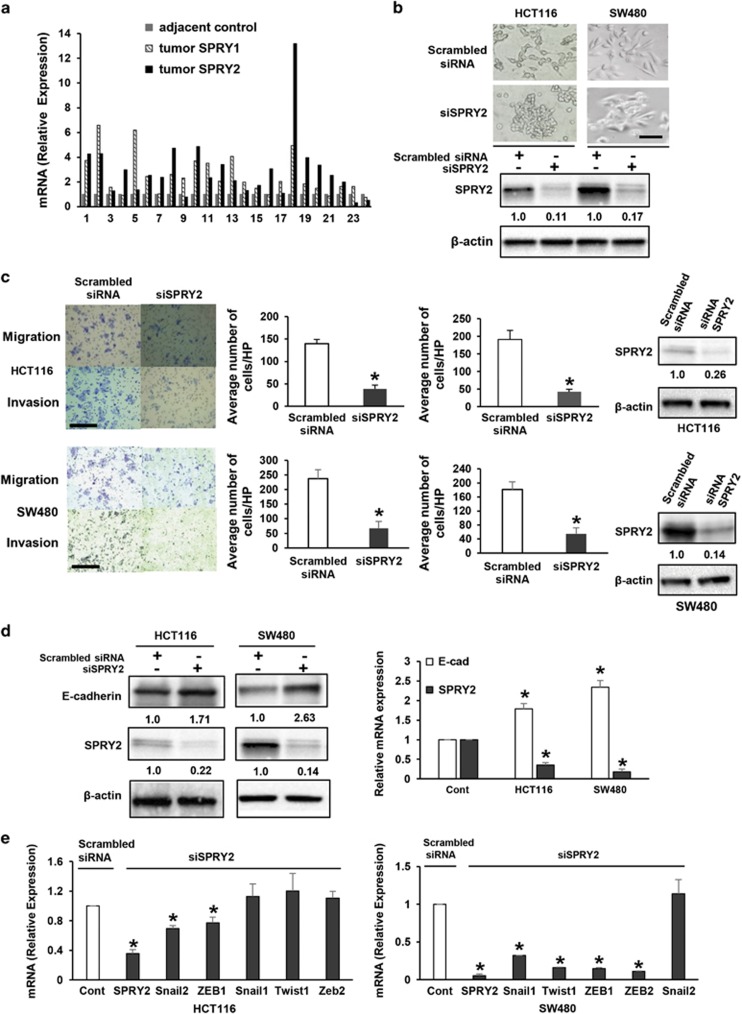
SPRY1 and SPRY2 expression is upregulated in human CRC and silencing of SPRY2 suppresses EMT in colon cancer cells. (**a**) SPRY1 and SPRY2 mRNA levels in a human colon adenocarcinoma cDNA array containing 24 matched pairs (48 samples) covering 24 normal and 24 adenocarcinomas. Expression levels are shown relative to matched normal samples from all 24 patients. (**b**) Morphological changes in SPRY2 siRNA (100 nm, 96 h) transfected HCT116 and SW480 cells. A representative western blotting with quantification of SPRY2 expression in control scrambled siRNA or siRNA SPRY2-transfected cells, size bar=50 μm. Results were confirmed by repeating the experiment seven times (*n*=7). (**c**) SPRY2 suppression decreases cell migration and invasion of HCT116 and SW480 cells in a trans-well migration and invasion assay. Average number of cells from three random microscopic high power (HP) fields per insert were counted, size bar=200 μm. Results from three experiments (*n*=3) were analyzed, and data are expressed as mean±s.d., *P<0.05. Western blotting represents relative SPRY2 expression in HCT116 and SW480 cells used in migration and invasion assays. (**d**) SPRY2 suppression increases E-cadherin protein and mRNA expression. A representative western blotting and quantification of SPRY2 and E-cadherin protein in control scrambled siRNA or siRNA SPRY2-transfected cells. Results were confirmed by repeating the experiment five times (*n*=5). SPRY2 and E-cadherin mRNA expression (mean±s.d., (*n*=5), *P<0.05) in HCT116 and SW480 cells transfected with control scrambled siRNA or siRNA SPRY2. (**e**) SPRY2 suppression decreases EMT markers (EMT-inducing transcription factors); mRNA levels were determined in HCT116 and SW480 cells transfected with control scrambled siRNA or SPRY2 siRNA. Results are mean±s.d., (*n*=5), *P<0.05.

We evaluated relative expression of SPRY1 and SPRY2 mRNA transcripts in human colon cancer tissues and adjacent controls by utilizing a colon cancer cDNA array. Majority of the cancer samples demonstrated upregulation of SPRY1 and SPRY2 transcripts as compared with adjacent controls (Figure 1a). To determine whether SPRY2 regulates EMT in colon cancer cells, we selected HCT116 and SW480 human colon cancer cell lines that contained different levels of endogenous SPRY2 protein expression (Figure 1b). SPRY2 downregulation by siRNA resulted in trans-differentiation of cancer cells and significantly shifted cell morphology from fibroblastoid or more-elongated fibroblast-like shape to epithelial-like shape in both cell lines irrespective of the endogenous level of SPRY2 expression (Figure 1b). In Boyden chambers, SPRY2 suppression significantly decreased cell migratory and invasive capabilities of HCT116 cells (Figure 1c). Similarly, an inhibition in cell migration and invasion was also observed in SPRY2-downregulated SW480 cells (Figure 1c). Multiple studies have shown that E-cadherin is intricately involved in retaining cell-to-cell contacts and inhibiting cell motility in cancer cells.^11^ To determine whether E-cadherin was affected by SPRY2, we assessed E-cadherin protein and mRNA expression in SPRY2-downregulated cancer cells. Suppression of SPRY2 significantly increased E-cadherin protein (1.7- and 2.6-fold in HCT116 and SW480 cells, respectively, *P*<0.05) and mRNA expression (2- and 2.5-fold in HCT116 and SW480 cells, respectively, *P*<0.05) in both cell lines (Figure 1d). Repression of E-cadherin in epithelial cells is mediated by EMT markers/EMT-inducing transcription factors SNAIL1, SLUG (SNAIL2), TWIST1 and ZEB1/2.^12, 13^ SPRY2 suppression significantly decreased SNAIL2 and ZEB1 in HCT116 cells, whereas SNAIL1, TWIST1 and ZEB2 were not significantly changed. However, in SW480 cells, SPRY2 suppression resulted in reduced SNAIL1, ZEB1/2 and TWIST1 expression, whereas SNAIL2 was unaffected (Figure 1e). Collectively, results indicate that SPRY2 may affect CRC metastasis in a positive manner and reduction of SPRY2 inhibits EMT in colon cancer cells.

### Inverse correlation of miR-194-5p with SPRY2 expression

Various MicroRNAs (miRs) control EMT and cancer cell migration and invasion during tumor metastasis.^14^ We hypothesized that SPRY2 expression differentially regulate oncogenic and tumor-suppressor miRs and their target genes during CRC metastasis. Preliminary studies were conducted to determine the effect of SPRY2 suppression on the miR expression profile. HCT116 cells were stably transfected with three different SPRY2 shRNAs (shRNA 7522, shRNA 589, shRNA 588) using lentiviral expression system. Because of the random integration of the lentivirus into the host genome, varying levels of SPRY2 gene knockdown was expected in puromycin-resistant colonies. Colonies infected with shRNA 7522 demonstrated maximum suppression of SPRY2 followed by shRNAs 589 and 588 (Supplementary Figure S1). Maximal suppression of SPRY2 in clone 7522 resulted in a more than twofold upregulation of miR-194-5p, -4423 and -3925 and downregulation of miR-21 and −491 (corresponding to at least ±1.0  log fold change) (Supplementary Table 1). Relatively lower suppression of SPRY2 in clone 589 caused lesser fold changes in the expression levels of above mentioned miRs. Furthermore, miRs were least affected, except miR-21, if SPRY2 was minimally suppressed (clone 588). These results demonstrate that a differential expression of certain miRs, depending upon the level of SPRY2 suppression, could be achieved in colon cancer cells. The role of oncogenic miR-21 in cancer including CRC is well established and has been studied extensively.^15^ We focused our investigation on miR-194, because this is mainly expressed in the gastrointestinal tract.^16^ The role of miR-4423, −3925 and −491 in CRC is not clearly understood at this point of investigation.

**Figure 2:**
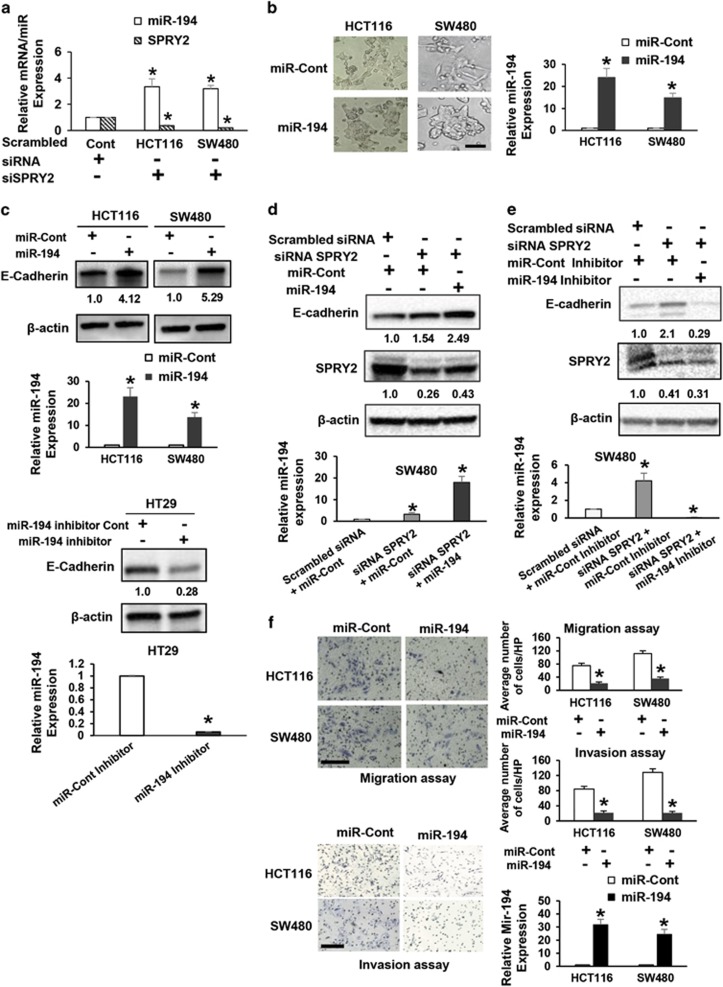
Increased miR-194 levels in SPRY2-downregulated cancer cells and miR-194 transfection suppresses EMT. (**a**) Cancer cells were transfected with scrambled siRNA or SPRY2 siRNA. SPRY2 suppression significantly increases miR-194-5p levels in HCT116 and SW480 cells. Results are mean±s.d. (*n*=5), *P<0.05. (**b**) Morphological changes in miR-194 mimic (100 nm, 96 h) transfected HCT116 and SW480 cells. The bar diagram represents relative miRNA-194 contents of miR-control or miR-194 mimic-transfected cells, size bar=50 μm. Results are mean±s.d. (*n*=5), *P<0.05. (**c**) miR-194 alters E-cadherin expression; a representative western blotting and quantification of E-cadherin protein in miR-control or miR-194 mimics (100 nm, 96 h) or miR-194 inhibitor (200 nm, 96 h) transfected cells (*n*=5). The bar diagrams represent relative miRNA-194 contents of miR-194 control or miR-194 mimic or inhibitor-transfected cells used for western blotting. Results are mean±S.D. (*n*=5), *P<0.05. (**d**) miR-194 mimic accentuates, whereas (**e**) miR-194 inhibitor decreases siSPRY2-induced upregulation of E-cadherin. A representative western blotting of E-cadherin and SPRY2 expression in SPRY2-downregulated SW480 cells or in combination with miR-194 mimic or miR-194 inhibitor transfections (*n*=3). The bar diagram represents relative miR-194 contents of miR-194 mimic or inhibitor-transfected cells used for western blotting. Results are mean±s.d. (*n*=3), *P<0.05. (**f**) miR-194 transfection decreases cell migration and invasion of HCT116 and SW480 cells in a trans-well assay. Average number of cells from three random microscopic fields per insert were counted, size bar=200 μm. Results from three independent experiments were analyzed, and data are expressed as mean±s.d., (*n*=3), *P<0.05. The bar diagram also represents relative miRNA-194 contents of miR-control or miR-194 mimic-transfected cells used in migration and invasion assays. Results are mean±s.d., (*n*=3), *P<0.05.

Mir-194 precursor is processed into miR-194-5p and miR-194-3p. Mir-194-5p is the major mature form of miR-194. To further confirm that SPRY2 suppression affects miR-194-5p expression levels, HCT116 and SW480 cells were transiently transfected with SPRY2 siRNA. MiR-194-5p levels were significantly upregulated (threefold, *P*<0.05) in HCT116 and SW480 cells (Figure 2a). Further, SPRY2 downregulation had no significant effect on miR-194-3p expression levels in cancer cells (Supplementary Figure S2). We then investigated endogenous contents of miR-194-5p in three different colon cancer cell lines that express different levels of SPRY2. HCT116 and SW480 cancer cells have significantly lower contents of miR-194-5p than HT29 cells (Supplementary Figure S3). Therefore, in subsequent experiments HCT116 and SW480 cells were used for miR-194 mimic transfection, whereas HT29 cells were used for miR-194 inhibitor experiments. Collectively, above results demonstrate that SPRY2 suppression by two different methods significantly upregulated miR-194-5p contents of colon cancer cells.

### MiR-194 negatively regulates EMT and colon cancer cell migration and invasion

As discussed above, SPRY2 downregulation significantly increased miR-194-5p levels, altered cancer cell morphology and decreased cell migration and invasion. Therefore, we then investigated the effect of miR-194-5p on cancer cell migration and invasion. The highest efficiencies of miR-194 transfection were achieved at 96 h posttransfection at 100 nm final concentration (data not shown). MiR-194 mimic-transfected HCT116 and SW480 cells assumed a condensed and a cobblestone-like or epithelial morphology when compared with cells transfected with miR controls (Figure 2b). MiR-194 mimics significantly increased E-cadherin expression in HCT116 (4.1-fold, *P*<0.05) and SW480 cells (5.3-fold, *P*<0.05), whereas miR-194 inhibitor reduced E-cadherin levels in HT29 cells (>70%, *P*<0.05) as compared with control transfections (Figure 2c). Mir-194 mimic transfection also increased E-cadherin mRNA transcripts by more than twofolds in HCT116 and SW480 cells (Supplementary Figure S4). In order to confirm SPRY2's role on miR-194-induced regulation of E-cadherin, we assessed E-cadherin induction during SPRY2 downregulation alone or in combination with miR-194 transfection (Figure 2d). Combination experiment resulted in additional induction of E-cadherin, demonstrating that other unknown pathways are also involved in E-cadherin induction (Figure 2d). We further extended these experiments, and effect of mR-194 inhibitor on siSPRY2-dependent upregulation of E-cadherin was investigated (Figure 2e). A complete reversal of E-cadherin induction indicates that miR-194 is a major downstream effector of SPRY2-dependent upregulation of E-cadherin (Figure 2e). To evaluate the migratory and invasive capabilities of miR-194 mimic-transfected cells, we also performed a modified Boyden chamber assay. MiR-194 mimic transfection significantly blocked cell migratory and invasive abilities of HCT116 and SW480 cells (Figure 2f). As expected, miR-194 mimic transfection also delayed cell migration in SW480 cells in a wound-healing assay (Supplementary Figure S5). In wound-healing assay, HCT116 cells are not viable in a 6-day-long experiment with low serum-containing medium. In summary, studies demonstrate a significant role of miR-194 in negative regulation of EMT in CRC.

### AKT2 is a direct target of miR-194

**Figure 3:**
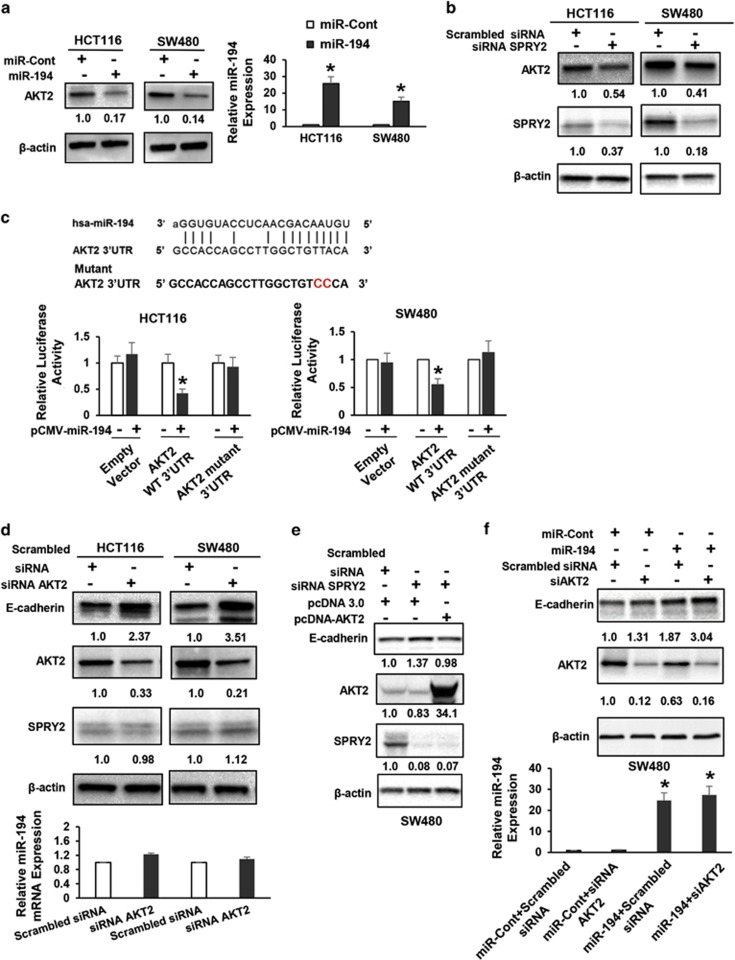
MiR-194 reduces AKT2. (**a**) miR-194 decreases AKT2 protein levels in cancer cells; a representative western blotting and quantification of AKT2 protein in miR-control or miR-194 mimic-transfected cells (*n*=5). The bar diagram represents relative miRNA-194 contents of miR-control or miR-194 mimic-transfected cells used for western blotting. Results are mean±s.d., (*n*=5), *P<0.05. (**b**) SPRY2 suppression decreases AKT2 expression. A representative western blotting of SPRY2 and AKT2 protein expression in HCT116 and SW480 cells transfected with control scrambled siRNA or siRNA SPRY2 (*n*=5). (**c**) Effect of miR-194 on the luciferase activities of wild-type and mutant AKT2 3′UTR reporters; cancer cells were transfected with pCMV miR-empty vector or miR-194 precursor vector (pCMV-miR-194) and pmir-GLO empty vector (Empty Vector) or 3′UTR wild-type AKT2 (pmir-GLO vector-AKT2 3′UTR) or 3′UTR mutant AKT2 (pmir-GLO vector-AKT2 3′UTR mutant) for 48 h. Luciferase activities were determined using a dual luciferase assay. Relative expression of firefly luciferase was standardized to a transfection control. Data represent mean±s.d. of three independent experiments in triplicate, *P<0.05. (**d**) AKT2 suppression increases E-cadherin expression; a representative western blotting and quantification of E-cadherin and AKT2 in HCT116 and SW480 cells transfected with control scrambled siRNA or AKT2 siRNA (*n*=5). Bar diagram represents miR-194 contents of control siRNA or siRNA AKT2 transfected cells. (**e**) AKT2 lacking 3′UTR transfection reverses siSPRY2-induced upregulation of E-cadherin. A representative western blotting of E-cadherin expression in SW480 cells transfected with siSPRY2 or in combination with AKT2 lacking 3′UTR (*n*=3). (**f**) miR-194 accentuates siAKT2-induced upregulation of E-cadherin. A representative western blotting of E-cadherin expression in SW480 cells transfected with siAKT2 or miR-194 mimics or in combination (*n*=3). The bar diagram represents relative miR-194 contents of control or siRNA AKT2 or miR-194 or combination of siAKT2 and mir-194-transfected cells used for western blotting (*n*=3). Data represents (one-way analysis of variance) mean±s.d. of three independent experiments, *P<0.05.

More often, miRs negatively regulate gene expression at the posttranscriptional level. The binding of miRs to complimentary sites in the 3′ untranslated regions (UTRs) of mRNA sequences cause either degradation of mRNA or inhibition of translation. Several genes of interest were predicted by the search programs of miRanda and TargetScan, and the effect of miR-194 on Talin2, SOX5, Musashi, AKT2, Rac1, HBEGF and IGF-1 R was tested by quantitative real-time (RT–PCR) and western blotting (Supplementary Figure S6). Majority of the targets were not affected; however, Talin-2 protein was downregulated. Further, Rac1 protein in HCT116 cells (Supplementary Figure S6A) and IGF-1 R mRNA in SW480 cells (Supplementary Figure S6B) showed increased steady-state levels, reflecting either indirect effects or atypical miR–target interactions.^17^ We observed a consistent significant downregulation of AKT2 expression (>80%, *P*<0.05) with predicted binding to 3′UTR of AKT2 in both cell lines (Figure 3a). Mir-194-dependent regulation of AKT2 has been recently demonstrated.^18^ We further tested whether SPRY2 suppression had any effect on AKT2 levels. Suppression of SPRY2 also decreased AKT2 expression though to a lesser extent when compared with miR-194 effects (>40%, *P*<0.05) (Figure 3b). To test whether AKT2 is a direct target gene of miR-194, we used a dual luciferase reporter, which has a conserved sequence of mRNA that is complimentary to the seed sequence of miR-194. Cells were co-transfected with miR-194 plasmid or empty vector and AKT2 wild-type 3′UTR or AKT2 mutant 3′UTR. MiR-194 significantly decreased luciferase activity in AKT2 wild-type 3′UTR transfected cells. Conversely, the luciferase activity of the mutant reporter was not affected in response to miR-194 plasmid treatment (Figure 3c). The results demonstrate that miR-194 may negatively regulate AKT2 posttranscriptionally in HCT116 and SW480 cells. A recent report has demonstrated that interaction of AKT2 with SNAIL1 in the E-cadherin promoter suppresses E-cadherin expression.^19^ We, therefore, extended our studies to assess E-cadherin repressors such as SNAIL1 and SNAIL2 that are not the direct targets of miR-194 as predicted by miR databases (picTar, miRanda and TargetScan). A significant downregulation of SNAILs was also observed in miR-194 mimic-transfected HCT116 cells (Supplementary Figure S7). The miR-194-dependent downregulation of AKT2 and SNAILs may account for increased expression of E-cadherin in these studies. In miR-194-transfected cells, downregulation of E-cadherin repressors other than AKT2 may represent an indirect regulation of E-cadherin repressors by miR-194 without any involvement of 3′UTR of these genes. We then hypothesized that AKT2 could also regulate EMT in colon cancer cells. Indeed, suppression of AKT2 significantly increased E-cadherin expression in both cell lines (Figure 3d). As expected, AKT2 suppression also delayed cell migration in a wound-healing assay in SW480 cells (Supplementary Figure S8). To confirm siSPRY's role on AKT-dependent upregulation of E-cadherin, we assessed E-cadherin induction during SPRY2 downregulation alone or in combination with the transfection of AKT2 plasmid lacking 3′UTR (Figure 3e). Combination experiment resulted in reversal of E-cadherin induction, demonstrating that AKT2 is an important player in E-cadherin induction by SPRY2 suppression (Figure 3e). To further confirm miR-194's role on AKT2's induced upregulation of E-cadherin, we assessed E-cadherin induction with siAKT2 or miR-194 transfection or in combination (Figure 3f). As expected, siAKT2 upregulated E-cadherin expression. Further, miR-194 transfection also induced E-cadherin expression to a greater extent as compared with siAKT2 response only. Furthermore, combination of siAKT2 and miR-194 accentuated E-cadherin expression when compared with siAKT2 or miR-194 responses alone (Figure 3f). Results demonstrate that miR-194 also affects targets, other than AKT2, by direct or indirect mechanisms involved in E-cadherin induction.

### MiR-194 altered E-cadherin localization in colon cancer cells

**Figure 4:**
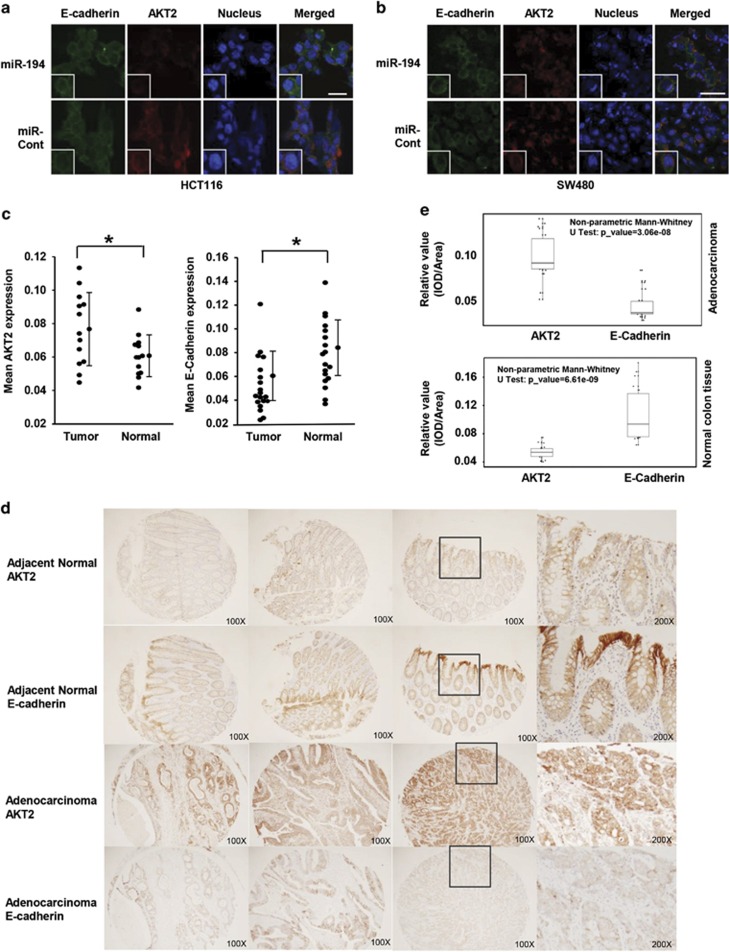
Mir-194 increases E-cadherin association with membrane and an inverse expression pattern of AKT2 with E-cadherin in human CRC. Cancer cells were transfected with miR-control or miR-194 and plated on collagen-coated cover slips. Immunofluorescence confocal microscopy was performed to assess the expression and localization of AKT2 and E-cadherin. Mir-194 transfection significantly increased membrane localization of E-cadherin and partial association of E-cadherin with AKT2 in (**a**) HCT116 and (**b**) SW480 cells, (*n*=5). The size bar is 20 μm. (**c**) Human CRC tissue microarrays were used to quantify AKT2 and E-cadherin expression. Tissue microarray contained paraffin-embedded sections from 33 matched pairs (66 samples covering 33 adjacent normal and 33 adenocarcinomas). To quantify immunostaining, images of cross-sections were captured under identical imaging conditions and imported into Image-Pro Plus software. Six regions of interest (ROI) were drawn. The amount of positive immunostaining within each ROI (expressed as positive pixels/mm^2^) was determined by false color segmentation analysis. Results are mean±s.d., *P<0.05. (**d**) Immunostaining of AKT2 and E-cadherin in three representative adenocarcinomas and adjacent normal tissue. (**e**) Boxplot charts demonstrating higher E-cadherin and lower AKT2 expression in normal adjacent tissue when compared with adenocarcinomas shown in panel (**d**). Immunohistochemical results were shown as Integral Optical Density (IOD)/area. Median value of each group is shown as the middle solid line within each box. Top and bottom lines of each box indicate 75th and 25th percentiles, respectively. Trendline connects the mean value of each group. Nonparametric Mann–Whitney *U*-test is adopted owing to the non-normal distribution of data. Both tests lead to significant differences at *P*<0.001.

We further validated our results by confocal microscopy and demonstrated a significant upregulation and redistribution of E-cadherin in miR-194-transfected HCT116 (Figure 4a) and SW480 (Figure 4b) cells. Cancer cells were transfected with miR-control or miR-194 mimics, and transfection was confirmed by increased miR-194 levels as assessed by RT–PCR (Supplementary Figure S9). Following the miR-194 transfection, cancer cells exhibit more E-cadherin immunoreactivity compared with control-transfected cells. Control cells show diffuse E-cadherin distribution throughout the cell. In miR-194-transfected cells, the immunoreactive E-cadherin appears in small vesicles that have a tendency to concentrate in the cell periphery in the area of plasma membrane. Furthermore, AKT2 immunoreactivity declined when cells were transfected with miR-194 as compared with control. AKT2-dependent oncogenic pathways can be activated through its translocation to the nucleus.^20^ However, miR-194 transfection moderately increased the association of AKT2 with E-cadherin on the cell membrane, but no nuclear translocation of AKT2 was noted. Collectively, data indicate that miR-194 may inhibit EMT by increasing expression and membrane localization of E-cadherin and partial association of E-cadherin with AKT2.

### Inverse expression pattern of AKT2 and E-cadherin in human colon cancer

We then raised the question whether AKT2 and E-cadherin expression are related in human CRC and evaluated immunoexpression of these genes in two sets of colon tumor tissue microarray containing tissue sections from the same cores. As compared with adjacent normal tissues, a significant upregulation of AKT2 and suppression of E-cadherin expression in tumors was noted (Figure 4c). Further, strong membranous staining of E-cadherin and weak cytosolic AKT2 expression was found in adjacent normal tissues. However, a significant decrease in membranous E-cadherin and increase in nuclear AKT2 staining was noted in human adenocarcinomas (Figure 4d). Statistical analysis also revealed an inverse expression pattern between AKT2 and E-cadherin in human colon cancer tissues (Figure 4e).

### Concurrent suppression of SPRY1 and SPRY2 upregulates E-cadherin, epithelial markers and suppresses mesenchymal markers

**Figure 5:**
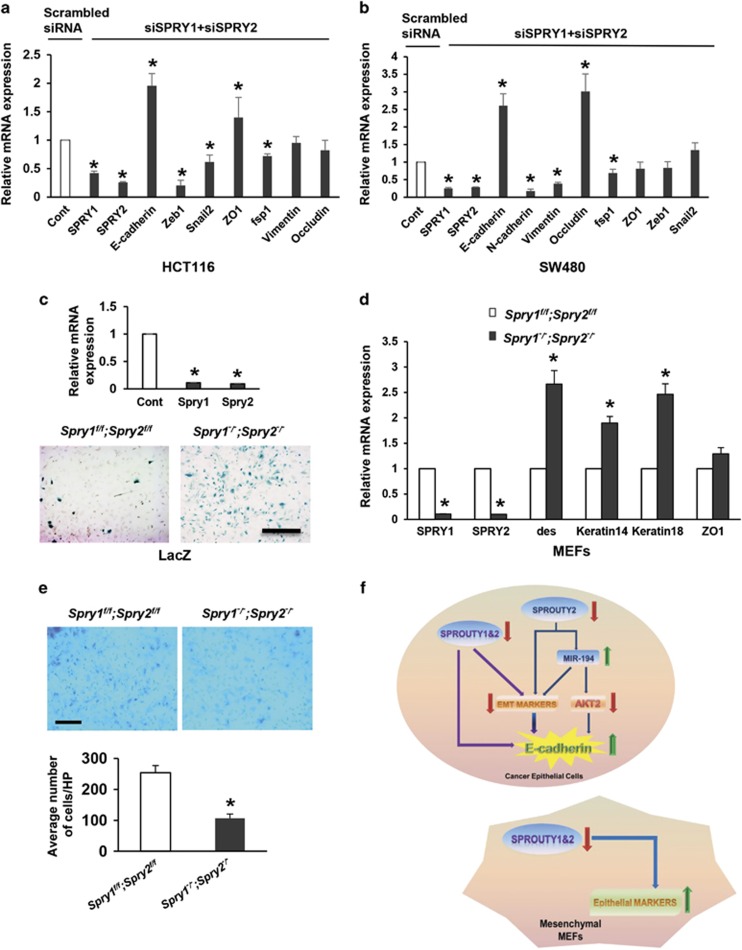
SPRY1 and SPRY2 suppression in colon cancer cells or deletion of *Spry1* and *Spry2* in MEFs upregulates epithelial and reduces mesenchymal markers. mRNA transcripts of epithelial (E-cadherin, ZO1 and Occludin) and mesenchymal/EMT (Zeb1, SNAIL2, fsp1, N-cadherin and Vimentin) markers in (**a**) HCT116 and (**b**) SW480 cells transfected with control scrambled siRNA or a mixture of SPRY1 (75 nm) and SPRY2 (75 nm) siRNA. Results are mean±s.d., (*n*=5), *P<0.05. (**c**) Tamoxifen treatment resulted in deletion of *Spry1* and *Spry2* that was confirmed by quantitative RT–PCR and lacZ expression, size bar=500 μm. Results are mean±s.d., (*n*=5), *P<0.05. (**d**) Tamoxifen-dependent recombination and deletion of *Spry1* and *Spry2* in MEFs increases the expression of epithelial markers. mRNA transcripts of des, ZO1, Keratin-14 and Keratin-18 in *Spry1*^*−/−*^*;Spry2*^*−/−*^ MEFs. Results are mean±s.d., (*n*=5), *P<0.05. (**e**) Deletion of *Spry1* and *Spry2* in MEFs reduces cell migration in a trans-well migration assay. Average number of cells from three random microscopic high power (HP) fields per insert were counted, size bar=400 μm. Results from three experiments (*n*=3) were analyzed, and data are expressed as mean±s.d., *P<0.05. (**f**) SPRY signaling in cancer cells and MEFs.

SPRY1 has also been implicated with tumorigenesis in prostate and breast cancer.^4^ SPRY1 and SPRY2 share a unique highly conserved COOH-terminal cysteine-rich domain. However, the NH2-terminal portion of SPRY protein is less conserved and exhibits only 25–37% identity among the different family members. These sequence differences could be responsible for the functional divergence among the SPRY proteins. We raised the question whether suppression of SPRY1 in colon cancer cells would exhibit a diverse effect on E-cadherin expression. Suppression of SPRY1 also increased E-cadherin expression in both cell lines (Supplementary Figure S10). We further raised the question whether suppression of both SPRY1 and SPRY2 in colon cancer cells would exhibit a diverse effect on mesenchymal/EMT markers. To test this hypothesis, we suppressed both SPRY1 and SPRY2 in cancer cells by siRNA method. Concurrent suppression of SPRY1 and SPRY2 also increased E-cadherin expression in HCT116 and SW480 cells (Figures 5a and b). Concurrent suppression accentuated the reduction of EMT markers (Figures 5a and b) as compared with SPRY2 suppression alone (Figure 1e). Further, SPRY1 and SPRY2 downregulation also increased epithelial marker ZO1 and suppressed mesenchymal marker fsp-1 and EMT markers Zeb-1 and SNAIL2 in HCT116 cells (Figure 5a). In SW480 cells, in addition to fsp-1 a significant decrease in mesenchymal markers Vimentin and N-cadherin and upregulation of epithelial protein Occludin was observed (Figure 5b). N-cadherin was not detected in HCT116 cells. Collectively, data indicate that suppression of both SPRY1 and SPRY2 had additional effects that favor epithelial phenotype in colon cancer cells.

### *Spry1*^*−/−*^*; Spry2*^*−/−*^ MEFs exhibited increased epithelial markers and reduced cell migration

We utilized SPRY1 and SPRY2 floxed mouse to evaluate biological significance of the loss of SPRY1 and SPRY2 in MEFs. *CAAG-CreER*^*TM*^*/+ Spry1*^*flox/flox*^*; Spry2*^*flox/flox*^*; ROSA26*^*lacZ*^*/+* MEF lines (here onwards referred as *Spry1*^*f/f*^*; Spry2*^*f/f*^) were cultured and treated with tamoxifen in MEF culture medium at 37 °C for 48 h to generate *CAAG-CreER*^*TM*^*/+ Spry1*^*−/−*^*; Spry2*^*−/−*^*; ROSA26*^*lacZ*^*/+* double mutant cells (here onwards referred as *Spry1*^*−/−*^*; Spry2*^*−/−*^). Addition of tamoxifen resulted in deletion of *Spry1* and *Spry2* that was confirmed by qRT–PCR and lacZ expression (Figure 5c). A complete recombination and >90% reduction in *Spry1* and *Spry2* transcripts was noted. Further, LacZ expression was present in >80% of cells after 48 h tamoxifen treatment (Figure 5c). MEFs were further incubated without tamoxifen in complete medium for 48 h, and mRNA contents of epithelial markers were assessed. MEFs exhibit the expression of variety of epithelial cell markers, including cytokeratins 14 and 18 as well as desmoplakin and ZO1.^21^ E-cadherin was not detected in these cells (data not shown). *Spry1*^*−/−*^*; Spry2*^*−/−*^ MEFs demonstrated a significant increase (*P*<0.05) in mRNA transcripts of desmoplakin, cytokeratins 14 and 18 when compared with *Spry1*^*f/f*^*; Spry2*^*f/f*^ MEFs (Figure 5d). Further, migration of *Spry1*^*−/−*^*; Spry2*^*−/−*^ MEFs across the migration chambers was significantly reduced (58%, *P*<0.05) as compared with *Spry1*^*f/f*^*; Spry2*^*f/f*^ MEFs (Figure 5e). Collectively, data indicate that SPRY1 and SPRY2 deletions commit embryonic MEFs to an epithelial status, which is highlighted by the increased expression of epithelial markers desmoplakin and cytokeratin and may be responsible for the decreased cell migration as observed in the current study.

## Discussion

The expression of SPRY in human CRC is a contentious issue and has not been explored extensively. We have demonstrated upregulation of SPRY2 protein in a small number of matched control CRC samples.^5^ Our results were supported by a study which showed upregulation of SPRY2 in undifferentiated high-grade tumors and at the invasive front of low-grade carcinomas.^9^ However, another study revealed reduced SPRY2 transcripts in human colon cancer.^7^ The reported differences in the level of SPRY2 transcript and protein may account for posttranscriptional and translational regulation of SPRY2 in CRC. Decreased mRNA levels in CRC could be ascribed to impaired transcription and/or increased degradation of the transcripts. Else, increased SPRY2 protein expression may suggest an increased mRNA translation and/or autonomous translation enhancing activity. Translation silencing is usually mediated by 3′UTR-mediated sequestration of the mRNA and/or miR-mediated inhibition of translation. Increased SPRY2 protein expression in CRC could be due to the loss of translation-silencing mechanisms and/or suppression of miRs that regulate 3′UTR of SPRY2. However, mechanisms that regulate SPRY2 expression in CRC require further investigation. Nonetheless, in the current investigation we noted upregulation of SPRY2 mRNA in a human CRC cDNA array that contained matched control samples.

We identified that knockdown of SPRY2 expression increases miR-194-5p contents in colon cancer cells. The role of miR-194 has been analyzed in normal and malignant cells of the gastrointestinal tract as high levels of miR-194 are expressed in the intestine and liver.^22, 23^ Reduced miR-194 expression was noted in CRC.^24^ An increased miR-194 expression in differentiating Caco-2 colon cancer cells has been reported.^25^ In this regard, we have noted decreased levels of SPRY2 during spontaneous Caco-2 cell differentiation (unpublished observation). In the present study, increased miR-194 expression in SPRY2-downregulated cells may indicate increased cancer cell differentiation. MiR-194 is transcriptionally regulated in intestinal epithelial cells.^26^ Hepatocyte nuclear factor has been reported to induce miR-194 expression during intestinal epithelial cell differentiation.^25^ Suppression of SPRY2 may positively regulate various transcription factors that bind to promoter for increased miR-194 transcription. Other intracellular factors may also regulate miR-194 expression in CRC. For example, methylation of miR-194-2 and -192 cluster promoter regulates miR-194 expression in cancer cells. Hypermethylation of this cluster promoter and epigenetic downregulation of miR-194 has been suggested in multiple myeloma.^27^ However, whether SPRY2 regulates transcription factors and/or methylation of miR-194 promoter is not clearly understood at this time and this supposition needs further investigation.

EMT, a key process contributing to tumor metastasis, is portrayed by the loss of the epithelial marker E-cadherin and increase in EMT- inducing transcription factors.^28^ E-cadherin, a cell–cell adhesion molecule, is functionally necessary for epithelial integrity.^29^ In CRC, loss of E-cadherin is associated with the loss of tumor differentiation, increased lymph node and distant metastasis. EMT-inducing transcription factors can repress E-cadherin directly or indirectly. Direct repressors include SNAIL superfamily and E-box-binding proteins of ZEB family. Factors such as TWIST and AKT repress E-cadherin transcription indirectly. AKT activation has been shown to induce SNAIL expression, which in turn represses E-cadherin.^30^ Further, TWIST transcriptionally upregulates AKT2 and represses E-cadherin expression.^31^ These studies clearly indicate that AKT and EMT-inducing transcription factors are interwoven in regulation of E-cadherin expression. We demonstrated that SPRY2 suppression alters cancer cell morphology from fibroblastoid to epithelial type and decreases cell migration with concomitant increase in E-cadherin expression and reduction in EMT-inducing transcription factors. Further, ectopic expression of miR-194 also augmented E-cadherin expression and lowered AKT2 expression by targeting 3′UTR of AKT2. In this regard, AKT2 upregulation has been shown to be associated with malignant transformation and EMT in CRC.^32^ AKT2 amplification is responsible for decreased apoptosis, increased survival, migration, invasion and metastasis in CRC.^20^ An important finding of our study was the inverse expression pattern of AKT2 and E-cadherin in human colorectal carcinomas. Interestingly, *in vitro*, studies also demonstrated that AKT2 suppression upregulates E-cadherin expression.

We established that concurrent suppression of SPRY1 and SPRY2 may have additional effects in decreasing EMT markers or EMT-inducing transcription factors and thus favoring epithelial phenotype in colon cancer cells. We extended our studies to MEFs that were isolated from the *Spry1* and *Spry2* floxed mouse. *In vitro*, deletion of *Spry1* and *Spry2* in MEFs augmented epithelial markers and decreased cell migration. It is not clear, however, at this point of investigation whether a similar phenotype would be present, *in vivo*, in colon epithelial cells of this model. In this regard, SPRY2 blocked fibroblast growth factor-induced mesonephric cell migration to the developing testis in male sex organogenesis.^33^ On the contrary, conditional deletion of SPRY1 and SPRY2 reduced motility in the ocular surface epithelial cells that results in 'EOB' (eyes open at birth) phenotype.^34^

In summary, our results have identified that suppression of SPRY leads to reduction in EMT and enhancement in a phenotype that favors epithelial features (Figure 5f). Suppression of SPRY upregulates E-cadherin/epithelial markers and suppresses EMT/mesenchymal markers in CRC cell lines. However, different cell lines showed different epithelial and EMT markers changed following SPRY suppression. Colon cancer cell lines differ with respect to many genetic derangements. This is reflected by their different growth rates and susceptibilities to various drugs. It is thus not surprising that two different cell lines will differ in response to alterations in SPRY expression as these cell lines likely also differ in SPRY substrates and SPRY regulators.^35^ SPRY2-dependent regulation of E-cadherin is the end-organ effect of reduction of both direct and indirect targets of miR-194. MiRs exert their end-organ effects via direct mRNA targets and by secondary and tertiary effects that accumulate through so-called 'indirect' targets. Cancer, a heterogeneous disease, cannot be successfully treated by targeting a single gene of interest. The promising ability of miRs to control several genes associated with a particular cellular pathway therefore may hold the key to therapeutic success. Thus regulation of miR-194 by SPRY2 and suppression of AKT2 and other EMT markers by miR-194 could be considered as a potential anticancer therapeutic strategy. We have earlier established a role of SPRY2 in maintaining oncogenic c-Met expression in CRC.^5^ Our findings were recently confirmed and a similar regulation of c-Met by SPRY2 has been reported in non-small cell lung cancer.^36^ Together, our studies indicate a role of SPRY in sustaining processes important to malignancy in CRC, and suppression of SPRY may inhibit EMT in CRC patients.

## Materials and methods

### Antibodies and RT–PCR primers

Supplementary Tables 2 and 3 describes antibodies and primers, respectively.

### Cell culture

Please see Supplementary Material.

### SPRY1 and SPRY2 mutant mouse

Female mice, between 3 and 4 months, carrying *Spry1*^*flox/flox*^,^37^ *Spry2*^*flox/flox*(^^ref. 38)^ and *ROSA26*^*lacZ*^*/ROSA26*^*lacZ*(^^ref. 39)^ alleles were crossed to B6.Cg-Tg (CAG-cre/Esr1)^5Amc/J^ male mice (CAAG-CreER, JAX stock number 004453)^40^ in which a ubiquitouly expressed Cre gene was fused to a tamoxifen-inducible mutant of the estrogen receptor. Cre-mediated recombination was initiated by incubation of MEFs in media containing 1μm tamoxifen, fixed in 1% glutaraldehyde and stained with X-gal as described. A complete method to generate MEFs is explained under Supplementary Material. All procedures were approved by the Institutional Animal Care and Use Committee of the Medical College of Wisconsin.

### SPRY2 suppression by lentiviral shRNA

Please see Supplementary Material.

### miR microarray profiling

RNA from SPRY2 shRNA or control non-silencing shRNA-infected cells were labeled using miRCURY Hy3/Hy5 Power Labeling Kit (Exiqon, Woburn, MA, USA). Hy3/Hy5 fluorescent-labeled RNA samples were hybridized to the miRCURYLNA LNA array (v.9.2) using a hybridization station. The quantified signals (background subtraction) were normalized using the global lowess (LOcally WEighted Scatterplot Smoothing) regression algorithm that utilizes within-slide normalization to minimize dye-dependent differences in intensity. Exiqon carried out the miRNA labeling, hybridization and preliminary array analyses. (GEO Repository http://www.ncbi.nlm.nih.gov/geo/query/acc.cgi?acc=GSE69665).

### RNA isolation

Please see Supplementary Material.

### Real-time PCR

Please see Supplementary Material.

### Transfection

Please see Supplementary Material.

### 3′UTR reporter assay

Briefly, cells were seeded in six-well plates, co-transfected with miR-194 precursor vector (p-CMV-miR-194) or empty vector control (Origene, Rockville, MD, USA) and a wild-type AKT2 3′UTR reporter construct (pmiR-GLO-AKT2-3′UTR) or pmir-GLO-AKT2-3′UTR mutant or empty vector with the firefly luciferase reporter and the control vector *Renilla* luciferase, pRL-TK (Promega, Madison, WI, USA) using lipofectamine 2000 following the manufacturer's protocol. *Renilla* luciferase vector (pRL-TK) served as an internal control and was included in all samples. After 48 h, cells were lysed and Firefly and Renilla activities were measured sequentially using the dual luciferase assay kit (PRE1910, Promega). Luminescence was measured by a luminometer (Turner model 9100-002, Turner Biosystems Inc., Sunnyvale, CA, USA).

### Confocal microscopy

Confocal microscopy using a Leica TCS SP8 confocal laser scanning microscope (Leica Microsystems Inc., Buffalo Grove, IL, USA) was performed as described earlier.^41^ A complete method is available under Supplementary Material.

### Western blotting

Western blotting was conducted essentially as described earlier.^5, 42^ Western blots were quantified by the ImageJ software (NIH, Bethesda, MD, USA). Values normalized to β-actin are indicated with control cells arbitrarily set as 1.0.

### Cell migration and invasion

Cell migration and invasion was conducted essentially as described earlier.^5^ Migration (07-200150) and invasion chambers (08-774122) were purchased from Corning (Tewksbury, MA, USA).

### Scratch (wound healing) assay

Please see Supplementary Material.

### Tissue microarray analysis

Formalin-fixed, paraffin-embedded human CRC tissue microarray (CO992 Biomax, Rockville, MD, USA) was utilized for AKT2 and E-cadherin immunostaining. A complete method is available under Supplementary Material.

### cDNA array analysis

Human 'TissueScan Colon Cancer cDNA Array' (HCRT103, Origene) was utilized for analysis of SPRY1 and SPRY2 mRNA expression. Specific RT primers were obtained from Qiagen (Rockville, MD, USA). PCR was performed in a thermocycler (Roche, Madison, WI, USA) using the qSTAR SYBR Master Mix Kit (Origene). SPRY1 and SPRY2 expression levels were normalized to those of β-actin using the ΔΔC_t_ method.

### Statistical analysis

Number of replicated experiments (*n*) and statistical tests used are indicated in the figure legends. Data were expressed as mean±s.d. One-way analysis of variance or an un-paired Student's *t*-test was used to evaluate statistically significant differences in the experimental group and the control group. *P*<0.05 was considered statistically significant. For non-normal distribution of data, nonparametric Mann–Whitney *U*-test was adopted. Sample size is not chosen to detect a prespecified effect, no randomization or inclusion/exclusion criteria were employed and all the data set is included for statistical analysis. Investigators were not blinded in cell culture experiments.
