# Erratum: Atypical role of sprouty in colorectal cancer: sprouty repression inhibits epithelial–mesenchymal transition

**DOI:** 10.1038/onc.2017.56

**Published:** 2017-03-13

**Authors:** Q Zhang, T Wei, K Shim, K Wright, K Xu, H L Palka-Hamblin, A Jurkevich, S Khare

**Keywords:** Intracellular signalling peptides and proteins, Epithelial-mesenchymal transition, Prognostic markers, Colorectal cancer

**Correction to:** *Oncogene* (2016) **35**, 3151–3162; doi:10.1038/onc.2015.365; published online 5 October 2015

**Updated online 13 March 2017:** This article was originally published under a CC BY-NC-ND 4.0 license, but has now been made available under a CC BY 4.0 license. The pdf and html versions of the paper have been modified accordingly.

