# Supplementary material for: Atypical role of sprouty in colorectal cancer: sprouty repression inhibits epithelial–mesenchymal transition
Source: Oncogene. 2015 Oct 5;35(24):3151–62. doi: 10.1038/onc.2015.365 (PMC4850112; doi:10.1038/onc.2015.365)
Supplement: Supplementary Information [file onc2015365x1.doc]

**Materials and Methods**

**Cell Culture**

Human colon cancer cell lines HCT116, SW480 and HT29 cells were purchased and maintained as described by the American Type Culture Collection (ATCC). Cell lines were authenticated by CellCheck 9 STR profiling (Human 9-Marker STR Profile and Inter-species Contamination Test). Further, mycoplasma contamination is routinely checked in the laboratory by utilizing a kit (Lonza, USA).

**SPRY1 and SPRY2 Mutant Mouse**

For individual embryos, head and viscera were removed. The remaining tissue was rinsed with PBS, minced, and digested with trypsin/versene (BioWhittaker, 17-161E) by stirring with glass beads for 30 min at 37°C. Digested cells were collected and resuspended in MEF culture medium (Dulbecco’s modified Eagle’s medium with 10% FBS, 0.1% NEAA, 2mM L-glutamine, 1mM sodium pyruvate, 50U/ml penicillin and 50μg/ml streptomycin). Embryos and MEF lines were genotyped to determine the presence of the *CAAG-CreER^TM^* allele and to confirm homozygosity of the *Spry1^flox^* and *Spry2^flox^* alleles. From each embryo approximately 5.0x10^6^ cells recovered, which were plated into 2 wells of a 6 well dish and grown to confluence and split.

**SPRY2 Suppression by Lentiviral shRNA**

SPRY2 knockdown was performed by infecting HCT116 cells with three different lentivirus expressing shRNAs and a control non targeted non-silencing shRNA (Sprouty2 MISSION shRNA Lentiviral Transduction Particles; TRCN 0000007522, TRCN 0000231589, TRCN 0000231588 and a non-targeted shRNA control from Sigma) by utilizing lentiviral transduction protocols provided by Sigma. Long term gene silencing was achieved by puromycin selection. HCT116 cells infected with lentivirus expressing control non-targeting shRNA were used as experimental controls.

**RNA Isolation**

Total RNA was extracted with RNA isolation kit (Exiqon, USA) according to the manufacturer's instructions and further purified using Turbo Kit (Ambion, USA). RNA purity was assessed by measuring absorptions at 260 and 280 nm and samples that had A260/A280 ratio of 1.9–2.1 were considered acceptable.

**Transfection**

Cancer cells (0.5 x 10^6^) were transfected by reverse transfection methods with miR-194 mimics (Dharmaco, 300642) or miR-194 inhibitor (Exiqon, 426899) or scrambled miRNA controls (Dharmacon, 001000), siSPRY1 and control non-silencing RNA (Origene, 306938 and 30004), siSPRY2 and control non-silencing RNA (SantaCruz, 41037 and 37007) siAKT2 and Control (Cell Signaling 6369S, 6568S) for 96 hr using Lipofectamine 2000 as described by the manufacturer (Invitrogen, USA).

**Real Time PCR**

For qRT-PCR analysis RT kits (Sigma and Bio-Rad, USA) were utilized. RNA was reverse transcribed with a cDNA kit (Bio-Rad, USA) into complementary DNA (cDNA) by utilizing specific qRT PCR primers in 20 μl total volume. cDNA was used as template for quantitative PCR in triplicate using SYBR green qPCR Mix (Roche, USA). Thermal cycling conditions were as follows: 95°C for 5 minutes, followed by 45 cycles as follows: denaturation step at 95°C for 10 sec, annealing step at 56°C for 10 sec and extension step at 72°C for 10 sec. U6 RNA was used as an internal control in Mir RT-PCR and GAPDH was used as an internal control for other genes. The ΔΔC_T_ method was used to compare the relative expression levels between treatments. The final PCR results were expressed as the relative expression compared to individual control sample in each assay.

**Confocal Microscopy**

Transfected cancer cells (0.5×10^4^) were seeded onto collagen (10µg/ml) coated glass coverslips. Cells were grown for additional 24 hours and fixed in ice-cold methanol for 15 min. Cells were permeabilized in 0.1% Tixton-100 in PBS for 3 min, blocked with 10% goat serum for 1 hr, incubated overnight at 4^o^C with rabbit anti-AKT2 (Abcam 66129, 1:200) or E-cadherin (SantaCruz 21791, 1:50) followed by incubation with secondary antibodies (1:200) conjugated with fluorescent dye CF^TM^488 (Biotium, 20302) and CF^TM^543 (Biotium, 20300) respectively. Far-red dot TM2 (Biotium, 40061) was used to reveal nuclear staining.

**Scratch (Wound Healing) Assay**

Cancer cells (5×10^5^ cells/well) were seeded to 90% confluence in a six-well plate. After desired transfections, a scratch was made through the center of each well using a pipette tip creating an open “scratch” or “wound” that was clear of cells. The dislodged cells were removed by three washes with culture media. Movement of cells in 2% serum containing was documented up to six days post-scratching using a Nikon inverted microscope.

**Tissue Micro Array Analysis**

Formalin-fixed, paraffin-embedded human CRC tissue micro array (CO992 Biomax, USA) was utilized for AKT2 and E-cadherin immunostaining. Sections were dewaxed, rehydrated using an alcohol gradient and heat-retrieved. Endogenous peroxidase activity was blocked using 0.3% H2O2/phosphate-buffered saline for 10 min at room temperature. The blocking buffer of the VECTASTAIN Elite ABC Kit (VECTOR Laboratories, USA) was used to block nonspecific binding sites. Sections were incubated with a polyclonal ani-AKT2 (1:200, Abcam) and anti-E-cadherin (1:400, Cell Signaling) overnight at 4^0^C. Subsequently sections were treated with anti-goat biotinylated secondary antibodies (1:200) and developed using diaminobenzidine as substrate (DAKO System). For negative controls, primary antibodies were omitted or sections were incubated with isotype-matched non-immune antibodies. Negative control sections showed no specific staining.

Supplementary Table 1

Lentiviral SPRY2 shRNAs (shRNA 7522, shRNA 589, shRNA 588) mediated suppression of SPRY2 differentially regulates miRs in HCT116 cells.

| **Hsa-miR** | **shRNA7522** | **shRNA589** | **shRNA588** |
| --- | --- | --- | --- |
| 194-5p | 1.271 | 0.446 | 0.041 |
| 3925-3p | 1.887 | 0.619 | 0.291 |
| 4423-3p | 1.553 | 0.610 | 0.357 |
| 21-5p | -1.054 | -0.429 | -0.590 |
| 491-3p | -1.086 | -0.4398 | -0.198 |

Log fold change of differentially expressed miRs in SPRY2 downregulated cells is shown. MiR expression profile analysis was done on miRCURYLNA^TM^ microarrays.

Supplementary Table 2

| **Antibody (Western Blots)** | **Vendor** | **Catalog number** | **Dilution** |
| --- | --- | --- | --- |
| Sprouty2 | Sigma | S1444 | 1:500 |
| Sprouty1 | Oncogene | TA305701 | 1:250 |
| E-cadherin | Cell signaling | 3195 | 1:1000 |
| AKT1 | Cell signaling | 2967 | 1:1000 |
| AKT2 | Cell signaling | 5239 | 1:1000 |
| Snail-1 | Cell signaling | 3879 | 1:1000 |
| Snail-2 | Cell signaling | 9585 | 1:1000 |
| HBEGF | Abcam | Ab92620 | 1:1000 |
| IGF1R | Cell signaling | 9750 | 1:1000 |
| Sox-5 | Santa Curz | Sc-20091 | 1:200 |
| Talin2 | Abcam | Ab108967 | 1:1000 |
| Musashi | Cell signaling | 2154 | 1:1000 |
| Rac1 | Cell signaling | 4651 | 1:1000 |
| β-actin | Cell signaling | 3700 | 1:3000 |
| **Antibody (Immunofluorescence)** | **Vendor** | **Catalog number** | **Dilution** |
| AKT2 | Abcam | 66129 | 1:200 |
| E-cadherin | Santa Cruz | 21791 | 1:50 |

Supplementary Table 3

Realtime PCR and cDNA array primers

| **Human** | **Forward** | **Reverse** |
| --- | --- | --- |
| Snail-1 | CGC GCT CTT TCC TCG TCA G | TCC CAG ATG AGC ATT GGC AG |
| Snail-2 | AAT ATG TGA GCC TGG GCG C | CTC TGT TGC AGT GAG GGC AAG |
| Zeb-1 | AGC AGT GAA AGA GAA GGG AAT GC | GGT CCT CTT CAG GTG CCT CAG |
| Zeb-2 | ACC AGC CCT TTA GGA GTT | AGA CCG ACA GGC GGA ATA |
| Twist1 | CGG GAG TCC GCA GTC TTA | GCT TGA GGG TCT GAA TCT TG |
| N-cadherin | CAC TGC TCA GGA CCC AGA T | TAA GCC GAG TGA TGG TCC |
| Vimentin | TGTCCAAATCGATGTGGATGTTTC | TTGTACCATTCTTCTGCCTCCTG |
| ZO-1 | TTT ATT TGG GCT GTG GCG TG | TTC CTC CAT TGC TGT GCT CT |
| β-catenin | GAG CCT GCC ATC TGT GCT CT | ACG CAA AGG TGC ATG ATT TG |
| Fsp1 | TCTTTCTTGGTTTGATCCTG | GCATCAAGCACGTGTCTGAA |
| GAPDH | TGC ACC ACC AAC TGC TTA GC | GGC ATG GAC TGT GGT CAT GAG |
| Talin2 | CAA TCT TGT TCG TGC AGC CCA GAA GG | GGC CGG TAT GTC CAG TTC TTG TTG GG |
| Musashi | GTT TCG GCT TCG TCA CTT TC | AAG GCC ACC TTA GGG TCA AT |
| Rac1 | CCA GAT GCA GGC CAT CAA GT | GCA GGC AGG TTT TAC CAA CAG |
| IGF1R | CGA CAT TGA GGA GGT CAC AGA | TGG GCA CGA AGA TGG AGT T |
| HBEGF | CTT TCT GGC TGC AGT TCT CTC G | GCC CCT TGC CTT TCT TCT TTC |
| SPRY2 (PPH08189E, Qiagen) | | |
| SPRY1 (realtimePCR.com, VHPS-8832) | | |
| AKT2 (realtimePCR.com, VHPS-283) | | |
| E-cadherin (realtimePCR.com, VHPS-1738) | | |
| Sox-5 (realtimePCR.com, VHPS-8763) | | |
| Mir-194-5p (Sigma, MIRAP00234) | | |
| Mir-194-3p (Sigma, MIRAP00235) | | |
| β-actin (Origene, cDNA array kit) | | |
| **Mouse** | **Forward** | **Reverse** |
| Des | TAC ACC TCA GGG CTG GAA AC | GGG CCA GTC TTA GCT CCT CT |
| FSP1 | GCT GCC CAG ATA AGG AAC CC | TGC GAA GAA GCC AGA GTA AGG |
| K14 | CCC AAT TCT CCT CAT CCT CTC | TAG GGA CAA TAC AGG GGC TCT |
| K18 | GAC GCT GAG ACC ACA CT | TCC ATC TGT GCC TTG TAT |
| Gapdh | GTT GTC TCC TGC GAC TTC A | GGT CCA GGG TTT CTT A |

Supplementary Figure S1

**Suppression of SPRY2 by shRNA** HCT116 cells were infected with a control non targeted (NTG) non-silencing shRNA and three different lentivirus expressing shRNAs (7522, 589, 588) specific against human SPRY2 mRNA. A representative western blot with quantification of SPRY2 protein is shown.

Supplementary Figure S2

**Effect of SPRY2 silencing on miR-194-3p expression** Cancer cells were transfected with scrambled siRNA and SPRY2 siRNA (100nM, 96 hrs). RNA samples were extracted and RT-PCR analysis of miR-194-3p was performed.

Supplementary Figure S3

**Endogenous levels of SPRY2 and miR-194-5p in colorectal cancer cells** (A) Western blot analysis for endogenous expression of SPRY2 in HCT116, SW480 and HT29 cells (B) RT-PCR analysis for relative endogenous expression of miR-195-5p in HCT116, SW480 and HT29 cells.

Supplementary Figure S4

**Effect of MiR-194 transfection on E-cadherin transcripts** HCT116 (A) and SW480 (B) cells were transfected with miR-194 mimics or control and E-cadherin transcripts were measured at indicated times by RT-PCR. Data represents mean ± S.D. of three independent experiments, *p< 0.05. (C) Relative miR-194 contents of cancer cells at 48 hr time point.

Supplementary Figure S5

**MiR-194 transfection decreases cell migration in a scratch assay** (A) SW480 cells were transfected with miR-control or miR-194 mimics and grown to confluence. A scratch was made in the monolayer. Images were recorded on day 0, 2, 5 and 6. Size bar = 250 µm. (B) Relative miR-194 contents of cancer cells at day 6. *p< 0.05

Supplementary Figure S6

**Effect of miR-194 transfection on target genes** HCT116 and SW480 cells were transfected with miR-control or miR-194 mimics and analyzed for (A) protein expression by western blotting and (B) relative mRNA expression by RT-PCR. Data represents mean ± S.D. of three independent experiments, *p< 0.05.

Supplementary Figure S7

**Effect of miR-194 on Snail1 and Snail2** HCT116 cells were transfected with control miRNA or miR-194 mimics. (A) Representative western blot of Snail1 and Snail2 (n=3) (B) Relative miR-194 content of miR-control or Mir-194 mimics transfected cells used for western blotting. Data represents mean ± S.D. of three independent experiments, *p< 0.05.

Supplementary Figure S8

**Silencing of AKT2 decreases cell migration in a scratch assay** SW480 cells were transfected with scrambled siRNA and siAKT2 and grown to confluence. A scratch was made in the monolayer. Images were recorded on day 0, 2, 5 and 6. Size bar = 250 µm. (B) AKT2 expression by western blotting at day 6.

Supplementary Figure S9

**Relative miR-194 expression in cancer cells** HCT116 and SW480 cells were transfected with miR-control or miR-194 mimics and relative contents of miR-194 were assessed by RT PCR. Data represents mean ± S.D. of five independent experiments, *p< 0.05.

Supplementary Figure S10

**Effect of SPRY1 silencing on E-cadherin expression** SPRY1 suppression increases E-cadherin mRNA expression. Relative E-cadherin mRNA expression in HCT116 and SW480 cells transfected with control scrambled siRNA or siRNA SPRY1. Data represents mean ± S.D. of three independent experiments, *p< 0.05.
