# Supplementary figures and images for: Atypical role of sprouty in colorectal cancer: sprouty repression inhibits epithelial–mesenchymal transition
Source: Oncogene. 2015 Oct 5;35(24):3151–62. doi: 10.1038/onc.2015.365 (PMC4850112; doi:10.1038/onc.2015.365)

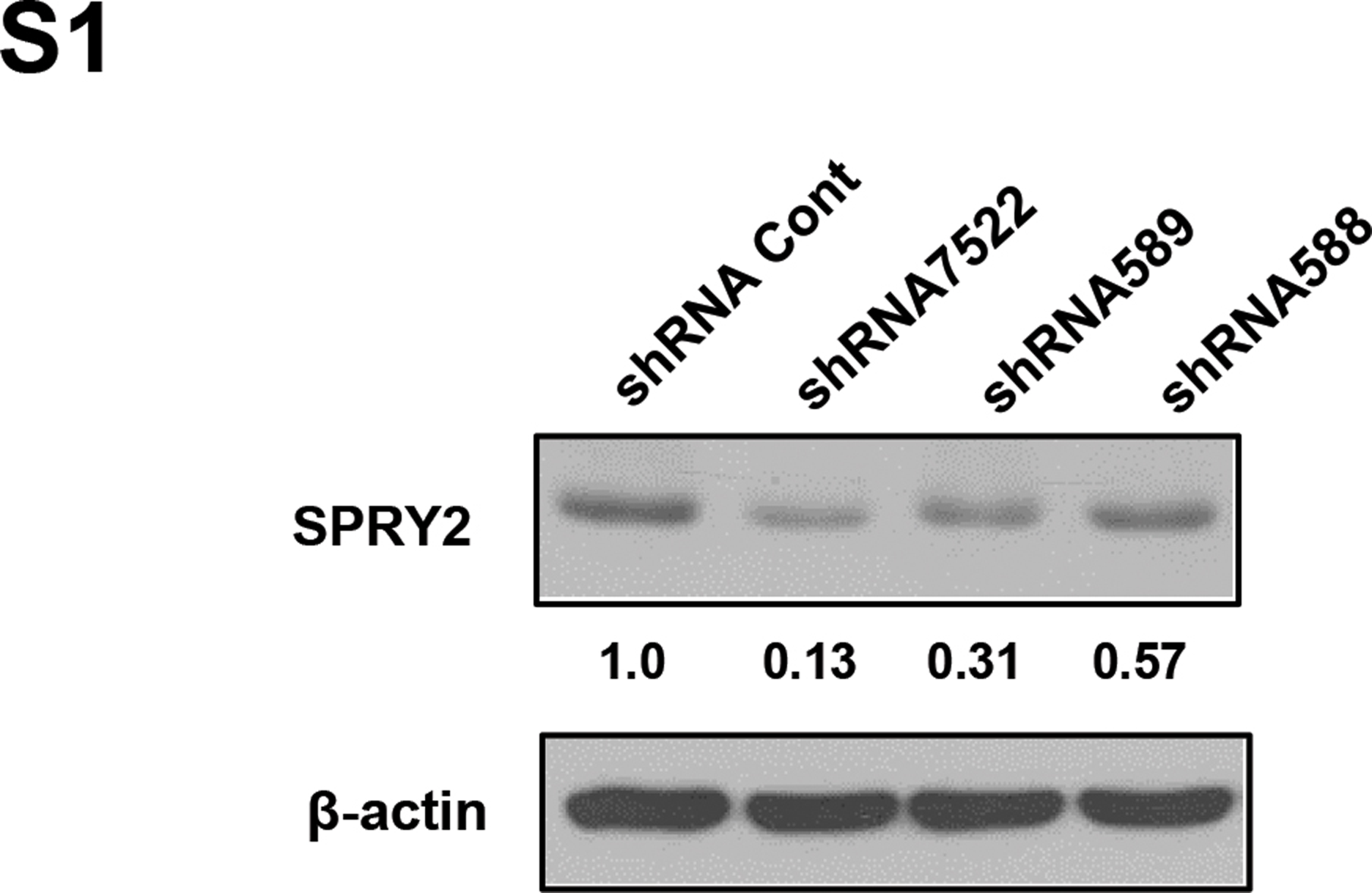

Supplement: Supplementary Figure 1 [file onc2015365x2.tif]

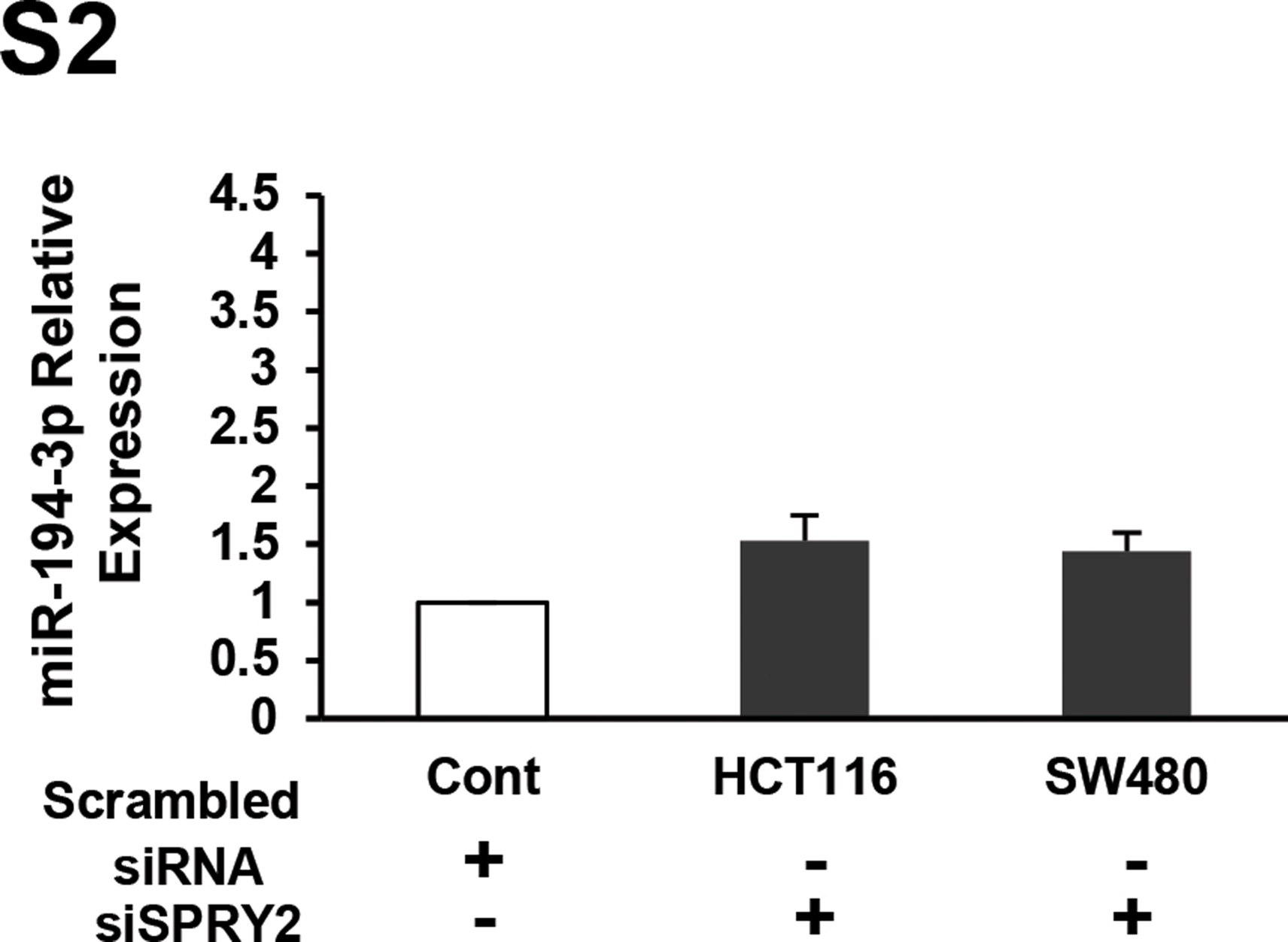

Supplement: Supplementary Figure 2 [file onc2015365x3.tif]

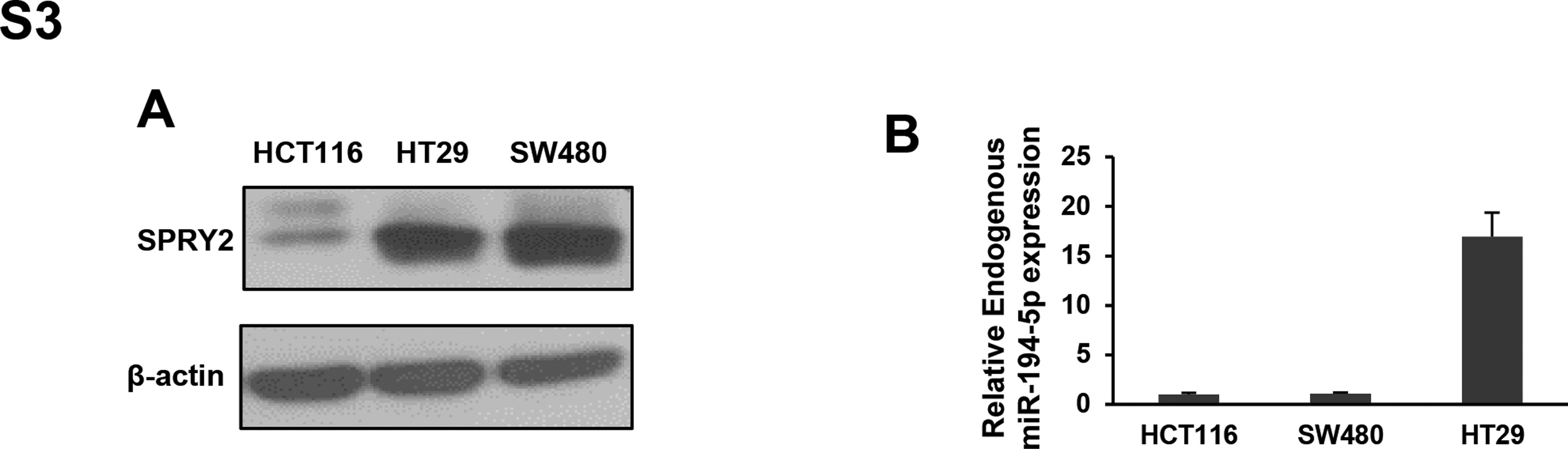

Supplement: Supplementary Figure 3 [file onc2015365x4.tif]

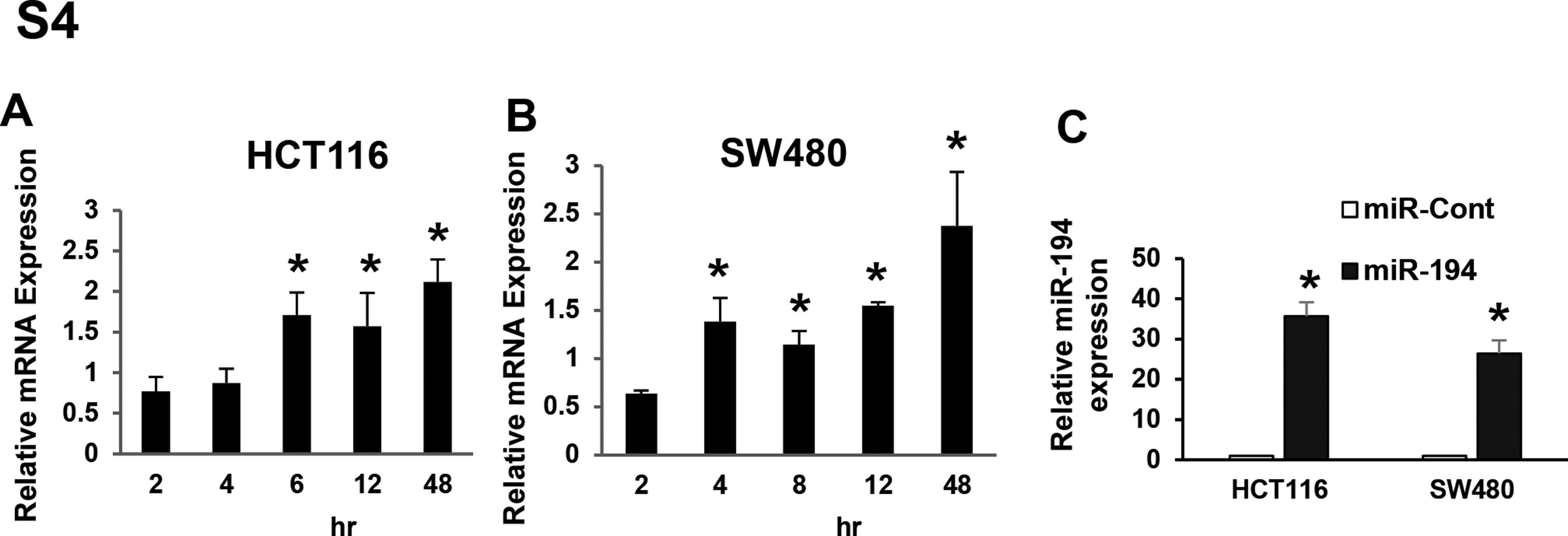

Supplement: Supplementary Figure 4 [file onc2015365x5.tif]

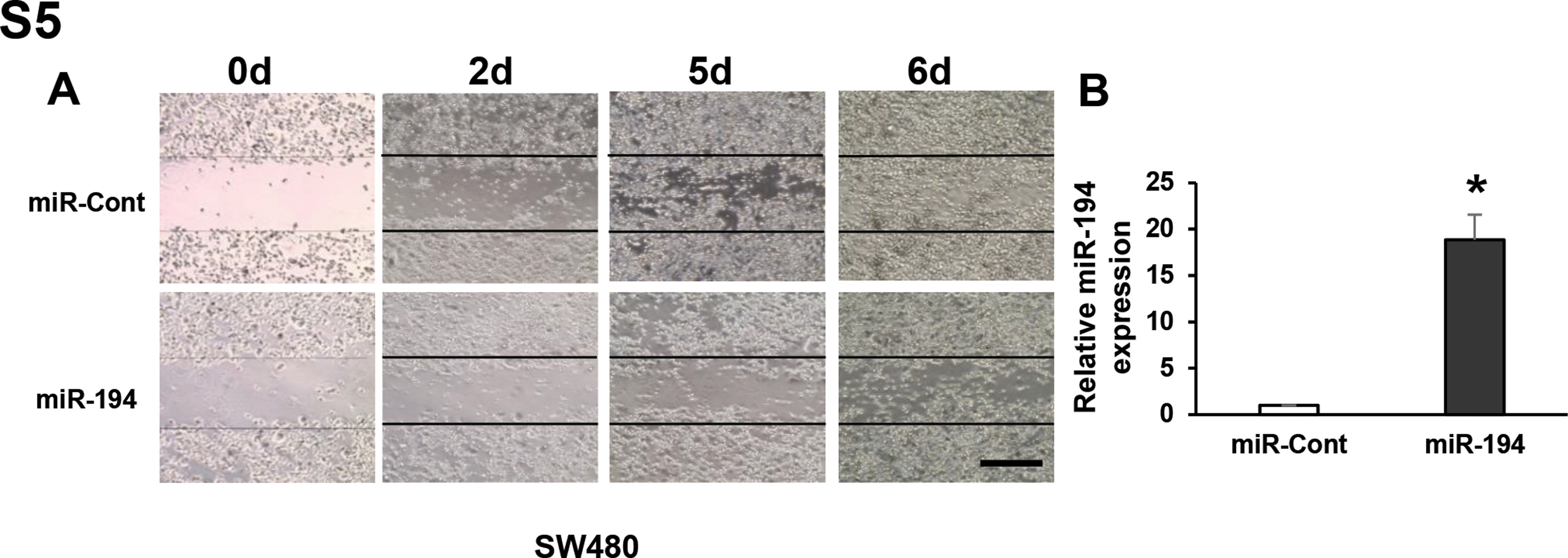

Supplement: Supplementary Figure 5 [file onc2015365x6.tif]

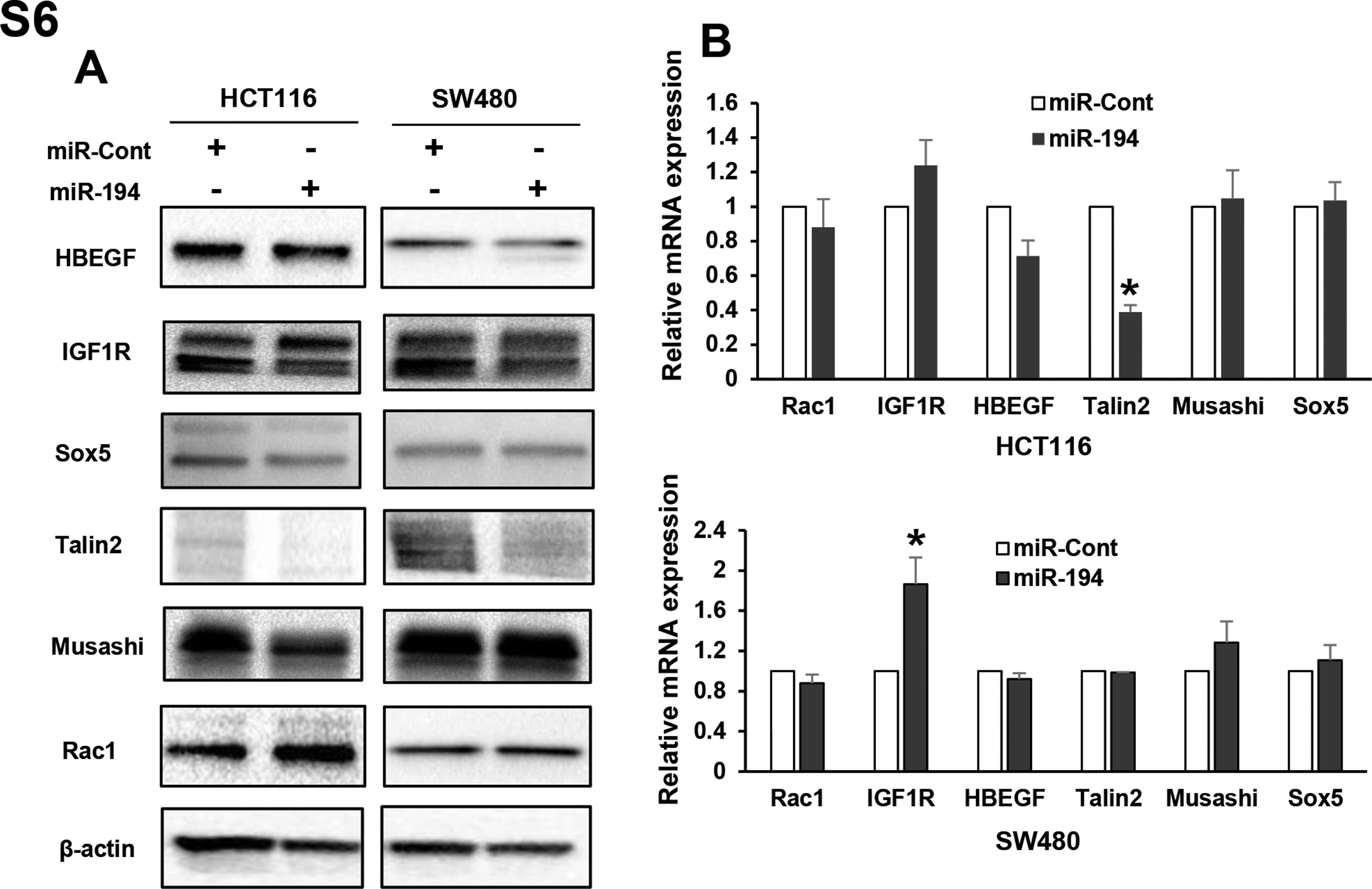

Supplement: Supplementary Figure 6 [file onc2015365x7.tif]

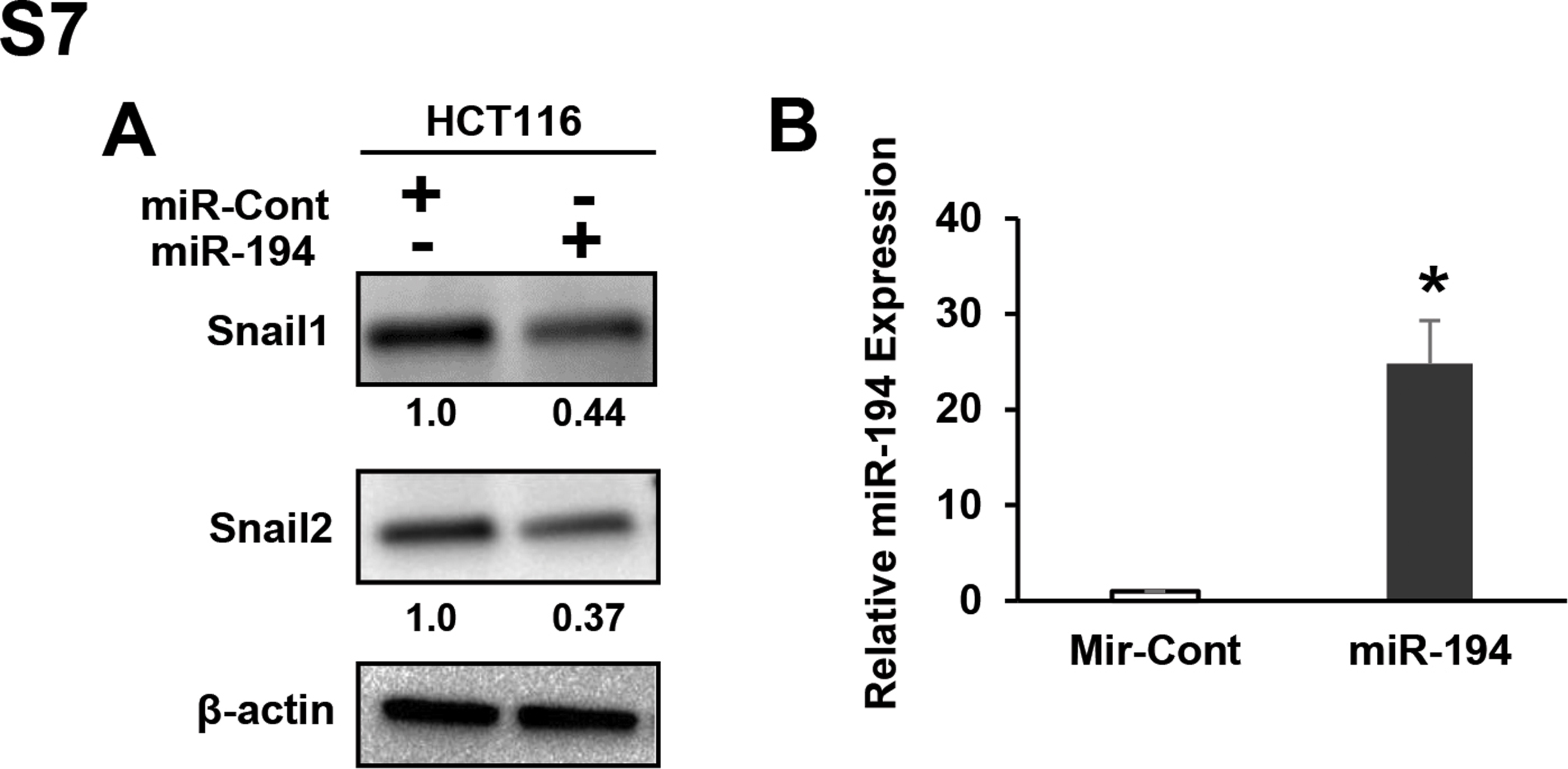

Supplement: Supplementary Figure 7 [file onc2015365x8.tif]

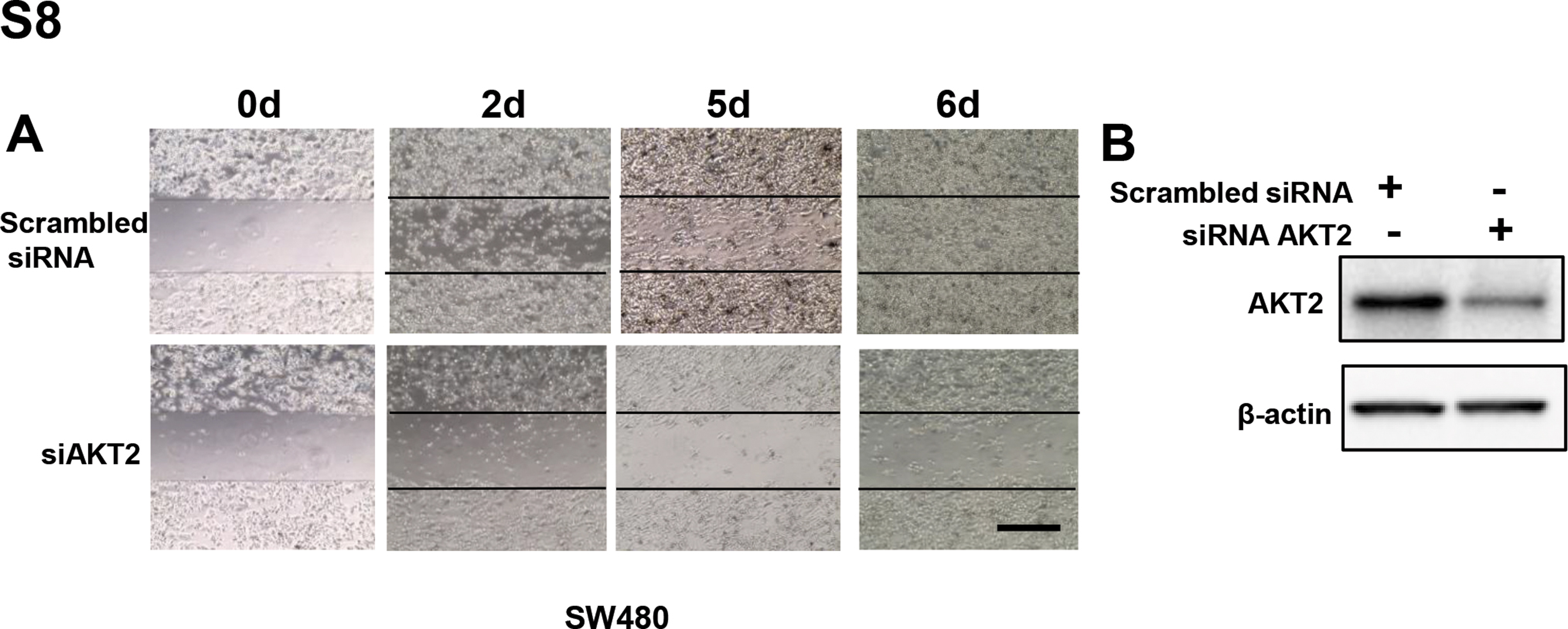

Supplement: Supplementary Figure 8 [file onc2015365x9.tif]

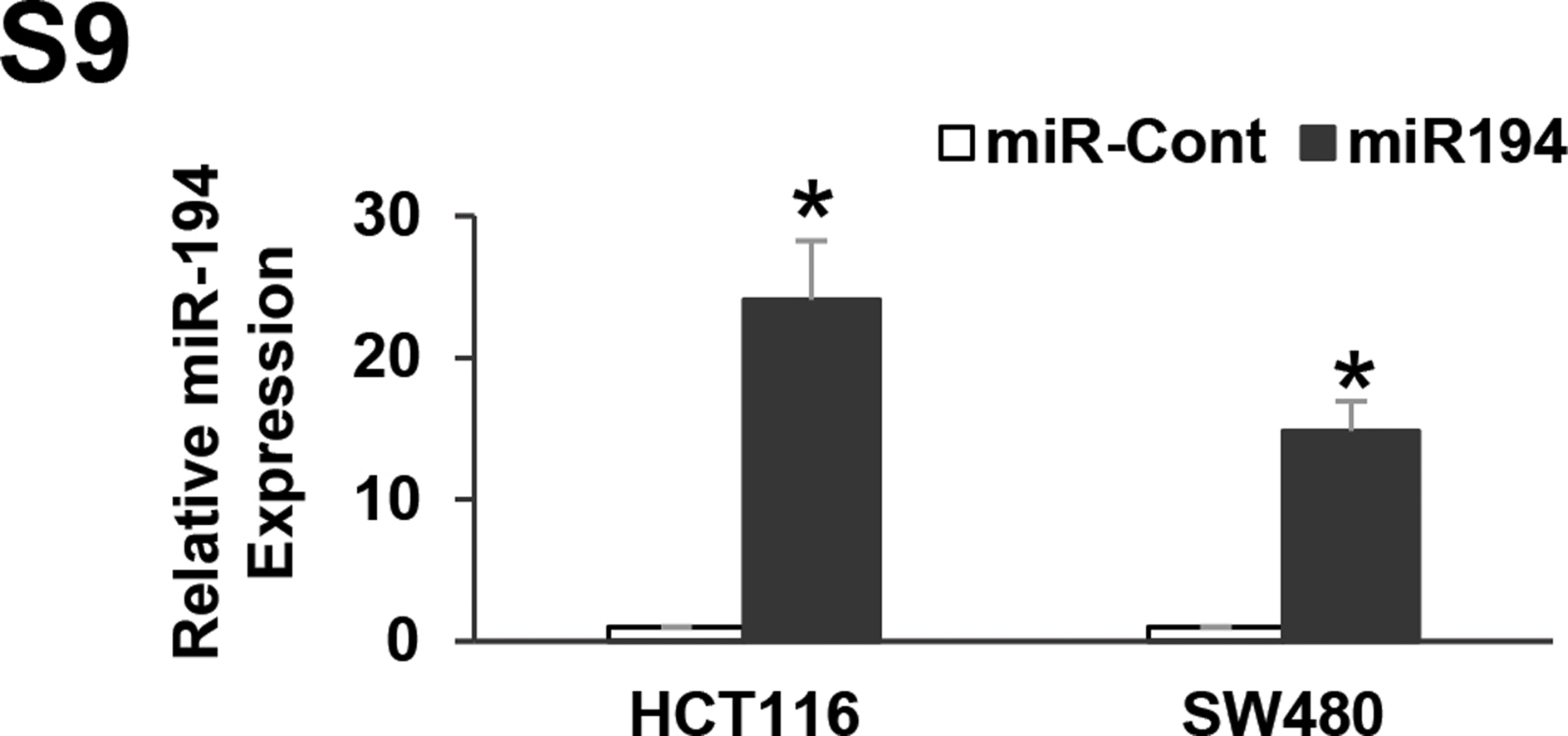

Supplement: Supplementary Figure 9 [file onc2015365x10.tif]

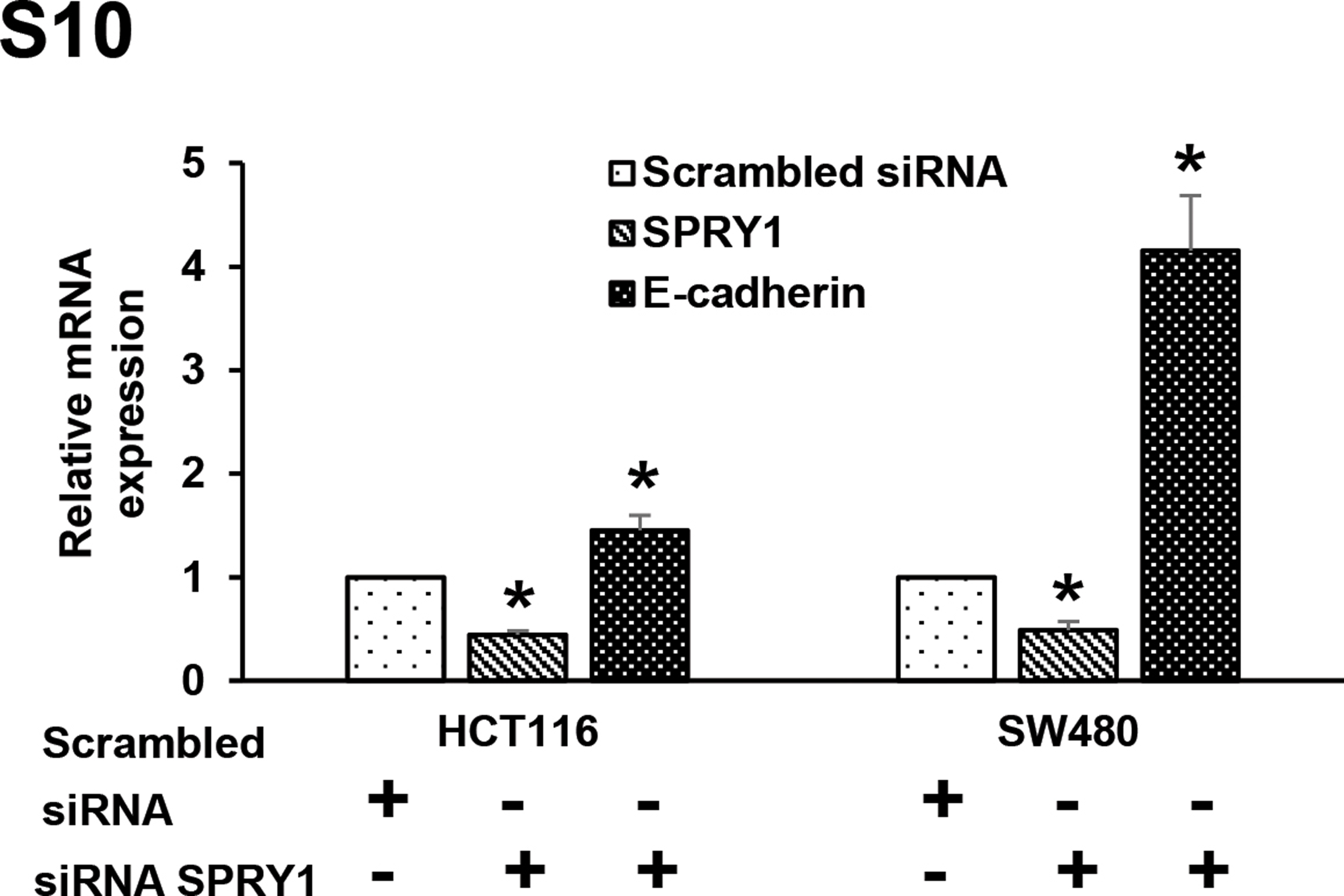

Supplement: Supplementary Figure 10 [file onc2015365x11.tif]
